# Human immunodeficiency virus type 1 ((HIV-1) subtypes in the northwest region, Cameroon

**DOI:** 10.1186/s12985-019-1209-6

**Published:** 2019-08-15

**Authors:** Lem Edith Abongwa, Anthony Kebira Nyamache, Judith Ndongo Torimiro, Paul Okemo, Fokunang Charles

**Affiliations:** 1 Department of Biochemistry, Biotechnology, and Microbiology, School of Pure and Applied Sciences, Kenyatta University, Nairobi, Kenya; 2 Department of Biological Sciences, Faculty of Science, University of Bamenda, Northwest, Region, Bamenda, Cameroon; 3 Laboratory of Molecular Biology, Chantal Biya International Center for Research on the Prevention and Management of HIV / AIDS (CIRCB), Yaounde, Cameroon; 4 Department of Biochemistry, Faculty of Medicine and Biomedical Science, University of Yaounde I, Yaounde, Cameroon; 5 Department of Pharmacotoxicology and Pharmacokinetics, Faculty of Medicine and Biomedical Sciences, University of Yaounde, Yaounde, Cameroon

**Keywords:** HIV-1, Genetic diversity, Northwest region, reverse transcriptase

## Abstract

**Background:**

The high genetic diversity of HIV-1 has been shown to influence the global distribution, disease progression, treatment success, and the development of an effective vaccine. Despite the low HIV prevalence in Cameroon, all the major HIV subtypes alongside several circulating recombinant forms (CRFs) and unique recombinant forms (URFs) have been reported in Cameroon. To date, HIV-1 diversity in some parts of Cameroon has been largely studied however, information on circulating HIV-1 subtypes in the Northwest region (NWR) of Cameroon is dearth. Therefore the aim of this study was to determine the current circulating HIV-1 subtypes among adults in the NWR of Cameroon.

**Methods:**

The genetic analysis of the reverse transcriptase region of the *po*l gene was performed on 81 samples. The samples were collected from drug naïve patients aged between 18 and 61 years residing within the rural and urban towns in the NWR during the period between February and April 2016. Viral RNA was extracted from plasma, reverse-transcribed, further amplified by nested-PCR before sequencing using an in-house protocol. Generated sequences were then phylogenetically analyzed together with references using MEGA 7.

**Results:**

Phylogenetic analysis revealed a broad viral diversity including CRF02 _AG (74.1%), F2 (7.4%), D (7.4%), G (3.7%), A1 (1.2%), CRF22_01A1 (2.5%), CRF06_cpx (1.2%), CRF09_cpx (1.2%), CRF11_cpx (1.2%). Three close epidemic clusters were found among F2 (1) and CRF02_AG (2) variants. For the first time we are reporting the CRF22_01A1 subtype in this region.

**Conclusion:**

Our findings update HIV-1 subtypes information in Cameroon and uphold previous studies that CRF02_AG is the most prevalent subtype. This CRF02_AG subtype may have important public health, research, and clinical consequences.

## Background

Human immunodeficiency virus (HIV) displays an extraordinary genetic diversity with four distinct groups: M (major), O (outlier), N (non M/O) and P (putative) with nine different subtypes (A[A1, A2, A3, A4], B, C, D, F[F1, F2], G, H, J, and K), and at least 98 circulating recombinant forms (CRFs) and multiple unique recombinant forms (URFs) within the major M Group [1]. These phylogenetic classifications are currently based either on nucleotide sequences derived from multiple subgenomic regions (*gag, pol* and *env*) of the same isolates or on full-length genome sequence analysis [2–4]. The genetic diversity and rapid variation of HIV-1 have shown to influence the spectrum of mutations that develop during selective drug pressure as well as complicate the development of effective vaccines [5–7].

The prevalence of the HIV infection in Cameroon has progressively dropped from 5.5% in 2007 [8] to 3.8% in 2016 [9]. In Cameroon, despite the low infection rate, previous studies have revealed circulation of all group M clades (A–D, F–H, J, K), CRFs and URFs. In addition, the CRFs include; CRF01_AE, CRF02_AG, CRF06_cpx, CRF09_cpx, CRF11_cpx, CRF13_cpx, CRF18_cpx, CRF22_01A1, CRF25_cpx, CRF36_cpx and CRF37_cpx have been detected with CRF02_AG being the most common CRFs causing infection in Cameroon [2, 10, 11]. Following this HIV-1 diversity and the presence of HIV-2, Cameroon remains an ideal Country to explore the genetic diversity of HIV-1 with a possible occurrence of diverse and unique CRFs.

Despite all this interest on HIV genetic diversity in this country, most studies have been conducted in urban and central parts of Cameroon (2, 10, 11). Studies involving other parts of the country like the NWR have not been adequately evaluated. The few studies undertaken in this region, even though sample sizes have been small (< 30 samples), have revealed a number of new strains [5, 12]. Due to the preexisting diverse HIV-1 subtypes, there is a likelihood of increasing mixed infections that could lead to the development of diverse circulating recombinant forms. Based on this trend, there is a need for surveillance of these diverse HIV subtypes, CRFs, and URFs that have implications on vaccine design and transmission fitness in this region. This study was therefore conducted to ascertain the current circulating HIV subtypes, CRFs, or URFs in the Northwest region of Cameroon.

## Materials and methods

### Study design and setting

A cross sectional study was conducted and five ART clinics systematically sampled; Bafut, Bali, Ndop and Santa District Hospitals and Bamenda Regional hospital of the NWR of Cameroon. Study participants were enrolled on the day of antiretroviral therapy (ART) initiation from February 2016 to April 2016. A standardized questionnaire and patient record file were used to collect demographic.

### Ethics statement

The National Ethics Committee of Cameroon approved the study protocols (NO2016/01/685/CE/CNERSH/SP). The purpose of the study and research procedures were fully explained to participants in the language (Pidgin English, English or French) best understood by the clients. Signed informed consent was obtained from each of the participants prior to the interviews and blood collection.

### Study participants

A total of 100 HIV-1 patients were enrolled from February 2016 to April 2016. The eligibility criteria were HIV-1 Highly active anti-retroviral therapy **(**HAART) naïve patients, male or female aged ≥18 years while the exclusion criteria were pre-exposure to HAART, and patients looking severely ill (unable to walk without support or sit up rightly). Of the 100 patients enrolled, 81(81%) samples were sequenced in required reverse transcriptase coding regions giving a successfully sequencing performance of 81%.

### CD4^+^ T cell counts

The CD4^+^ T cell counts was performed using a FACS Calibur flow cytometer (Becton-Dickinson, NJ) equipped with automated acquisition and analysis software according to the manufactures instructions [13].

### RNA extraction, polymerase chain reaction, and sequencing

Five milliliters of whole blood was drawn from each subject into an EDTA (Ethylene Diamine Tetra acetic Acid) tube and centrifuged at 3000 rpm for 5 min. Two plasma aliquots of 1 ml each were collected from each patient and stored at − 80 °C until transported in an ice box to the Molecular Biology Laboratory of CIRCB-Yaoundé for analysis at CIRCB-Yaoundé where the samples were stored at − 20 °C. Plasma Viral RNA was extracted from 1 ml plasma using the QIAamp Viral RNA Mini kit (Qiagen, Valencia, CA) according to the manufacturer’s instructions. Extracted RNA was transcribed to cDNA using in-house RT –PCR protocol using a one-step kit (SuperScript™One-Step RT-PCR System, Invitrogen, USA). Partial HIV-1 *pol*-RT gene corresponding to 2075–3703 bp (the position is given referring to theHXB2 strain) was amplified by nested PCR using in-house protocol. Of the 100 samples, 81(81%) were successful amplified, purified, and directly sequenced using ABI 3130 genetic analyzer (Applied Biosystems, Foster City, CA) according to previously published in-house protocol with the following primers as shown on Table 1 [14].

**Table 1:** List of primers

Reaction	Name	Sequences	Position
RT-PCR	BS	5’GAC AGG CTA ATT TTT TAG GG3′	Forward
	GIO2	5’TTT CCC CAT ATT ACT ATG CTT 3’	Reverse
Semi nested	BS	5’GAC AGG CTA ATT TTT TAG GG3’	Forward
	TAK 3	5′ GGC TCT TGA TAA ATT TGA TAT GT 3’	Reverse
Sequencing	B	5’CAG GAA TGG ATG GCC CAA AA3’	Forward
	F	5’CCA TCC ATT CCT GGC TTT AAT 3’	Reverse
	SEQ1	5’AGC AGA CCA GAG CCA ACA GC 3’	Forward
	SEQ2	5’ATT TTC CCT TCC TTT TCC ATT TC3’	Reverse
	SEQ3	5’TTG TAC AGA AAT GGA AAA GGA AGG 3’	Forward
	SEQ4	5’TTT GTT CTA TGC TGC CCT ATT TCT 3’	Reverse
	SEQ5	5’GGC AGC ATA GAA CAA AAA TAG AGG3’	Forward
	TAK 3	5′ GGC TCT TGA TAA ATT TGA TAT GT 3′	Reverse

### Phylogenetic analysis

**Fig. 1:**
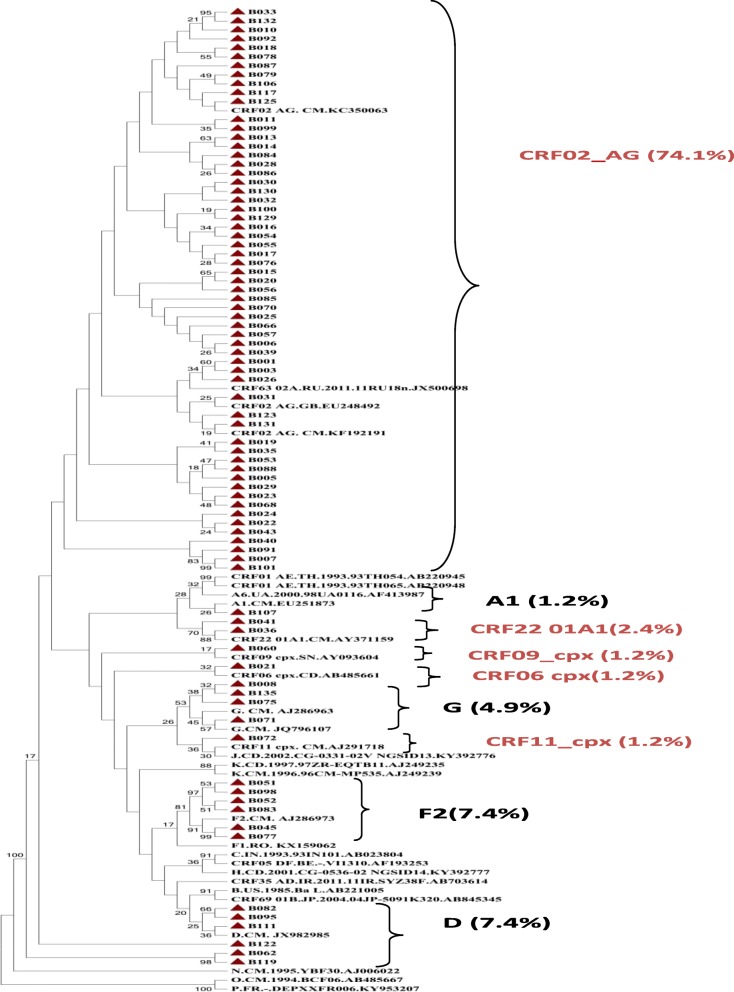
A Phylogenetic tree of HIV-1 pol gene. Sample sequences have red shapes. The sequences were aligned using ClustalW, and phylogenetic analysis performed using the neighbor-joining method of MEGA.v.7 software with Bootstrap value of 1000 replicates. The reference sequences were taken from the Los Alamos database (the subtype precedes the country letters code and reference accession number). Some references have been omitted to enable better visualization of the sample sequences. Abbreviation: CRF; circulating recombinant form, cpx; complex

Generated sequences were aligned using CLUSTAlW version 1.8.3 with subsequent inspection and manual modification with pair wise evolutionary distances estimated by Kimura’s two-parameter method with bootstrap analysis of 1000 replicates. Resulting trees were visualized using Fig tree software. Bootstrap resampling (1000 data sets) of multiple alignments was performed to test the statistical robustness of the trees (Fig. 1). Viral recombinants were confirmed using recombinant identification program (RIP) subtyping tool available at (http://www.hiv.lanl.gov/content/sequence/RIP/RIP.html).

### Drug resistance

HIV-1 genotypic drug resistance in the pol-RT region was defined as the presence of one or more resistance-related mutations as specified by the consensus mutation guidelines of the International AIDS Society-USA as well as consensus B subtypes https://hivdb.stanford.edu/hivdb/by-sequences/ which were used as reference strains for the definition of mutations [15].

### Data analysis

Statistical analysis was done using SPSS version 23 (Armonk, USA). Baseline characteristics of study patients were described using frequencies and percentages.

## Results

The participants were aged between 18 and 61 years, with a median age of 36.9 years and 55.5% (45) were female. The mean (range) CD4^+^ T cell count was 194.3 (8–498) cells/mm^3^ and a greater part of the participants had CD4^+^ T cells count of < 200 cells/mm^3^ 59.3% (48). The majority of the patients were residents in an Urban setting 54.3% (44) (Table 2).

**Table 2:** Descriptive characteristics of study participants (*n* = 81)

Indicator	Variable (n)	Female (%) *n* = 45	Male (%) *n* = 36
Site	Rural (37)	24(53.3)	13 (36.1)
	Urban (44)	21(46.7)	23 (63.9)
Age in years	Mean ± SEM	36.31 ± 1.41	37.39 ± 1.51
	Range	18–56	20–61
	< 30 (17)	12 (70.6)	5 (29.4)
	30–40 (37)	18 (48.8)	19(51.2)
	> 40 (27)	15 (55.6)	12(44.4)
CD4 Classification cells/ mm^3^	Mean ± SEM	207.36 ± 21.27	177.97 ± 20.84
	Range	8–489	31–498
	< 200 (48)	23 (49.9)	25 (52.1)
	200–350 (19)	13 (68.4)	6 (31.6)
	350–500 (14)	9 (64.3)	5 (35.7)

### Phylogenetic analysis

**Fig. 2:**
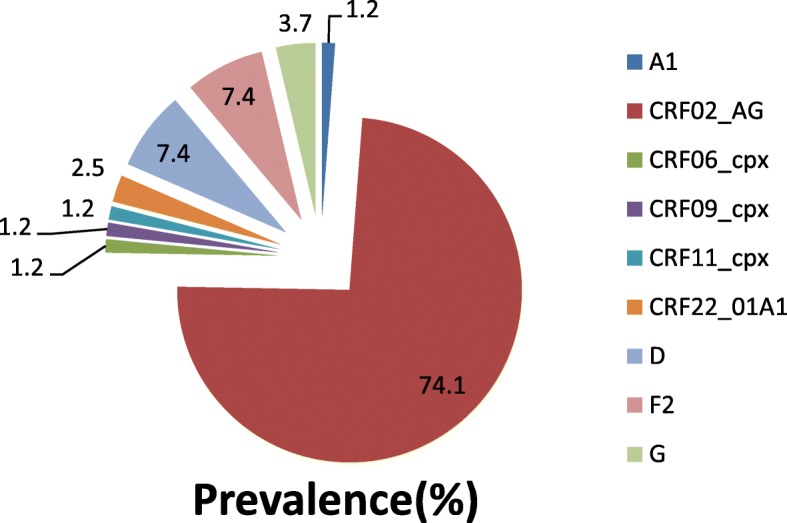
Graphic depiction of the distribution of HIV-1 variants. Abbreviations: CRF; circulating recombinant form, cpx; complex

From the 81 samples that were successfully amplified and sequenced, analysis of these sequences revealed; four pure subtypes and five CRF; F2 (6: 7.4%), G (4: 4.9%), D (3: 3.7%),A1 (1: 1.2%) pure subtypes and CRF02_AG (60: 74.1%), CRF22_01A1 (2: 2.4%), CRF06_cpx (1: 1.2%), CRF09_cpx (1: 1.2%) and CRF11_cpx (1: 1.2%) CRFs (Fig. 1). From this study CRF02_AG remains the most predominant circulating HIV-1 strains in Cameroon (Fig. 2). Although a small region of HIV-1 genome was amplified, new CRF in circulation in Cameroon, CRF22_01A1 (2.4%) was detected in this region.

### Drug resistance

This study shows that 9 (11.1%) patients were infected with HIV variants that carried resistance associated mutations associated with Nucleoside reverse transcriptase inhibitor (NRTI); 8.6% (7/81), Non nucleoside reverse transcriptase inhibitor (NNRTI); 4.9% (4/81) and protease inhibitors (PI); 1.2% (1/81). In this study, the most common mutations were K219Q (2.5%; 2/81) and E138A (2.5%; 2/81) which confers resistance to NRTIs and NNRTIs respectively. Singleton mutations that were associated with NRTI included: D67N, K70R, T215F, T215TA, M184MV, M184 V, D67N, K70R, K70 T, and M41ML; while that of, NNRTI was K103 N, V108I, V179E, and Y181C and for PI was I54IFV. Dual-class drug resistance mutation involving both NRTI and NNRTI was observed in three patients (3.7%).

## Discussion

Previous studies have shown that CRF02_AG is the dominant virus circulating in Cameroon. This study showed that CRF02_AG was the dominant HIV-1 subtypes in circulation involving 74.1% of the entire samples analyzed (Fig. 2). Consistent with findings from other regions in Cameroon, this recombinant remains to be the most dominant [10, 11, 16, 17]. Cameroon is known harbor diverse HIV-1 subtypes and groups [11, 17, 18]. By virtual of this circulating diverse HIV subtypes or CRFs, any transmission across populations has led to high viral recombinants and low pure subtypes, a situation that is being observed in Cameroon [10, 11, 16, 17].

The recombinant CRF02_AG which predominates (74.1%) in this study falls within the 48.6–80% range of previous studies conducted in Cameroon and other countries in West Africa, Central Africa, and Europe [2, 16, 19–21]. The high prevalence of CRF02_AG suggests that this viral strain may be well adapted in the Cameroonian population due to a founder effect from the parent strains subtype A and G, or may have some biological advantages such as a higher replicative fitness relative to parental subtype A and G which have been shown to have relatively higher replicative fitness in comparison to parental HIV-1 subtypes A and G which are known to circulate in West and East African countries [2, 10, 21]. Thus, the high prevalence of CRF02_AG species is suggestive that CRF02_AG will certainly aid in the design of an effective vaccine.

Compared with previous studies from different urban towns in Cameroon, the prevalent subtypes after CRF02_AG were F2, G, and D contrary to detected G, F2, and D [18] and D, F2, G subtypes [17]. The difference in levels of diverse subtypes in this region could be associated with the diverse disease burden in regions and population migrations from one region to another either due to political instability, occupation or commercial activities.

The CRF22_01A1 variant seen in this study with a very low prevalence has not been reported in the NWR [5, 12].The prevalence of this variant ranges from 1.6–13.9% from previous studies carried out in other regions of Limbe, Yaounde, Douala and Bertoua [10, 11, 17].This variant together with preceding data suggests that the CRF22_01A1 is not a new CRF strains but it might have been spread to this regions due to population migration from other regions or neighborhood countries [11, 17]. Introduction of this new HIV-1 variants in this region, confirms the occurrence of high levels of viral recombination that is being experienced in the country to existence of diverse HIV-1 subtypes in Cameroon.

Phylogenetic analysis of these sequences showed clusters with reference sequences from Cameroon and surrounding countries indicate possible local origins. This implies that transmissions of these viral strains are within the country.

Transmitted drug resistance (TDR) was however determined in this study. Overall prevalence of TDR was 11.1%. This prevalence was found to be higher compared to those detected (7.2%) in West and Central Africa in 2016 [22]. The moderate prevalence (5–15%) seen in the NWR could be attributed to poor drug adherence, loss of follow-up, pharmacy stock-outs, lack of proper patient retention in care and the use of ARV drugs to prevent mother to child transmission or as pre and post-exposure prophylaxis that leads to acquired drug resistance [23, 24].

The high prevalence of NRTI over NNRTI mutations detected was contrary to previous finding in other urban centres in Cameroon [25, 26]. However, these findings were similar to those obtained elsewhere by Vergne et al., [27] and Mbunkah et al.*,* [28]. This high level of NRTIs mutations detected in this study could be associated with the shift from the use of monotherapy NNRTI to the use of HAART among HIV infected pregnant and breast feeding women in the prevention of mother to child transmission of HIV [29, 30]. The detected NRTIs, K219Q mutation is known to confer resistance to Zidovudine (AZT) or Stavudine (D4T) [28]. Detection of this mutation could be associated with acquisition with already HIV drug resistant strains from infected individuals on either AZT or D4T treatment [31, 32]. Nevertheless, K103 N, V108I, V179E, and Y181C, NNRTI mutations that confer resistance to Efavirenz (EFV) and Nevirapine (NVP) were also detected [28, 33]. Based on the ART drug combinations, these mutations could similarly be associated with the transmission of resistant viral strains from already known populations on treatment. In this study, E138A mutation which is usually weakly selected in patients receiving second-generation NNRTIs (Rilpivirine and Etravirine) were also detected. However, detection of this mutation to these drugs could be associated with NNRTI’s low genetic barrier [26, 29]. The detected high prevalence of E138A mutations implies that it uses as salvage therapy could be limited without effective monitoring.

## Conclusions

Our data show an increasing diversity of HIV-1 in the NWR. This study confirmed previous findings that CRF02_AG subtype is still the most predominant subtype. In addition, the overall prevalence of TDR among recently diagnosed individuals in Northern region of Cameroun was moderate. We suggest that there is need for implementation of effective measures that could strengthen monitoring and guide ART usage and surveillance of HIV genetic diversity.

## Data Availability

The data sets used and analyzed during this study are available with the corresponding author on request. The DNA sequences of the HIV-1 protease-reverse transcriptase region of Pol that were determined in this study were submitted to GenBank under the following accession: MK061035-MK061115.

## Abbreviations

AZT: Zidovudine
Cpx: complex
CRF: circulating recombinant forms
D4T: Stavudine
ETR: Etravirine
HIV: human immunodeficiency virus
NNRTI: Non-nucleoside reverse transcriptase inhibitor
NRTI: Nucleoside reverse transcriptase inhibitor
NVP: Nevirapine
NWR: Northwest region
PCR: polymerase chain reaction
PR: protease
RNA: Ribonucleic acid
RT: reverse transcriptase
TDR: Transmitted drug resistance
URF: Unique recombinant forms

## References

[1] http://www .hiv.lanl.gov/content/sequence/HIV/CRFs/CRFs.html, Accessed 09 April 2019.

[2] Véras NM, Santoro MM, Gray RR, Tatem AJ, Presti AL, Olearo F (2011). Molecular epidemiology of HIV type 1 CRF02_AG in Cameroon and African patients living in Italy. AIDS Res Hum Retrovir.

[3] Patiño-Galindo JÁ, Torres-Puente M, Bracho MA, Alastrué I, Juan A, Navarro D (2017). The molecular epidemiology of HIV-1 in the Comunidad Valenciana (Spain): analysis of transmission clusters. Sci Rep.

[4] De Oliveira F, Mourez T, Vessiere A, Ngoupo PA, Alessandri-Gradt E, Simon F (2017). Multiple HIV-1/M+ HIV-1/O dual infections and new HIV-1/MO inter-group recombinant forms detected in Cameroon. Retrovirology..

[5] Tebit DM, Zekeng L, Kaptué L, Salminen M, Kräusslich HG, Herchenröder O (2002). Genotypic and phenotypic analysis of HIV type 1 primary isolates from western Cameroon. AIDS Res Hum Retrovir.

[6] Kantor R, Katzenstein DA, Efron B, Carvalho AP, Wynhoven B, Cane P (2005). Impact of HIV-1 subtype and antiretroviral therapy on protease and reverse transcriptase genotype: results of a global collaboration. PLoS Med.

[7] Santoro MM, Perno CF. HIV-1 genetic variability and clinical implications. ISRN microbiology. 2013;2013. 10.1155/2013/481314 PMC3703378 23844315

[8] Mbanya D, Sama M, Tchounwou P (2008). Current status of HIV/AIDS in Cameroon: how effective are control strategies?. Int J Environ Res Public Health.

[9] Cameroon population-based HIV impact assessment (CAMPHIA) 2017. https://phia.icap.columbia.edu/wp-content/uploads/2018/07/3471CAMPHIA_Cameroon-SS_A4_v13_requests_7.25.18.pdf Accessed 09 April 2019.

[10] Agyingi L, Mayr LM, Kinge T, Orock GE, Ngai J, Asaah B (2014). The evolution of HIV-1 group M genetic variability in southern Cameroon is characterized by several emerging recombinant forms of CRF02_AG and viruses with drug resistance mutations. J Med Virol.

[11] Teto G, Tagny CT, Mbanya D, Fonsah JY, Fokam J, Nchindap E (2017). Gag P2/NC and pol genetic diversity, polymorphism, and drug resistance mutations in HIV-1 CRF02_AG-and non-CRF02_AG-infected patients in Yaoundé. Cameroon Scientific reports.

[12] Burda ST, Konings FA, Williams CA, Anyangwe C, Nyambi PN (2004). HIV-1 CRF09_cpx circulates in the north West Province of Cameroon where CRF02_AG infections predominate and recombinant strains are common. AIDS Research & Human Retroviruses.

[13] Bosire EM, Nyamache AK, Gicheru MM, Khamadi SA, Lihana RW, Okoth V (2013). Population specific reference ranges of CD3, CD4 and CD8 lymphocyte subsets among healthy Kenyans. AIDS Res Ther.

[14] Fokam J, Salpini R, Santoro MM, Cento V, D’Arrigo R, Gori C (2011). Performance evaluation of an in-house human immunodeficiency virus type-1 protease-reverse transcriptase genotyping assay in Cameroon. Arch Virol.

[15] Nyamache AK, Muigai AW, Nganga Z, Khamadi SA (2012). HIV type 1 genetic diversity and naturally occurring polymorphisms in HIV type 1 Kenyan isolates: implications for integrase inhibitors. AIDS Res Hum Retrovir.

[16] Ceccarelli L, Salpini R, Moudourou S, Cento V, Santoro MM, Fokam J (2012). Characterization of drug resistance mutations in naïve and ART-treated patients infected with HIV-1 in Yaounde, Cameroon. J Med Virol.

[17] Courtney CR, Agyingi L, Fokou A, Christie S, Asaah B, Meli J (2016). Monitoring HIV-1 group M subtypes in Yaounde, Cameroon reveals broad genetic diversity and a novel CRF02_AG/F2 infection. AIDS Res Hum Retrovir.

[18] Ndembi N, Abraha A, Pilch H, Ichimura H, Mbanya D, Kaptue L (2008). Molecular characterization of human immunodeficiency virus type 1 (HIV-1) and HIV-2 in Yaounde Cameroon: evidence of major drug resistance mutations in newly diagnosed patients infected with subtypes other than subtype B. J Clin Microbiol.

[19] Negedu-Momoh OR, Olonitola OS, Odama LE, Inabo HI, Mbah HA, Kasembeli AN (2014). Antiretroviral-drug resistant mutations and genetic diversity in HIV-1 infected individuals in Nigeria. World Journal of AIDS.

[20] Carr JK, Wolfe ND, Torimiro JN, Tamoufe U, Mpoudi-Ngole E, Eyzaguirre L, Birx DL, McCutchan FE, Burke DS (2010). HIV-1 recombinants with multiple parental strains in low-prevalence, remote regions of Cameroon: evolutionary relics?. Retrovirology..

[21] Powell R, Barengolts D, Mayr L, Nyambi P (2010). The evolution of HIV-1 diversity in rural Cameroon and its implications in vaccine design and trials. Viruses..

[22] WHO, (2017). HIV drug resistance report 2017. Geneva. License, CC BY-NC-SA 3.0 IGO. https://www.who.int/hiv/pub/drugresistance/hivdr-report-2017/en/ Accessed 3^rd^ May 2018.

[23] Billong SC, Fokam J, Aghokeng AF, Milenge P, Kembou E, Abessouguie I (2013). Population-based monitoring of emerging HIV-1 drug resistance on antiretroviral therapy and associated factors in a sentinel site in Cameroon: low levels of resistance but poor programmatic performance. PLoS One.

[24] Fokam J, Elat JB, Billong SC, Kembou E, Nkwescheu AS, Obam NM (2015). Monitoring HIV drug resistance early warning indicators in Cameroon: a study following the revised World Health Organization recommendations. PLoS One.

[25] Fokam J, Bellocchi MC, Armenia D, Nanfack AJ, Carioti L, Continenza F (2018). Next-generation sequencing provides an added value in determining drug resistance and viral tropism in Cameroonian HIV-1 vertically infected children. Medicine..

[26] Tchouwa GF, Eymard-Duvernay S, Cournil A, Lamare N, Serrano L, Butel C, et al. Prevalence of pretreatment HIV drug resistance in Cameroon following a nationally representative WHO survey. J Antimicrob Chemother. 2018. 10.1093/jac/dky221 PMC11325248 29931063

[27] Vergne L, Diagbouga S, Kouanfack C, Aghokeng A, Butel C, Laurent C (2006). HIV-1 drug-resistance mutations among newly diagnosed patients before scaling-up programmes in Burkina Faso and Cameroon. Antivir Ther.

[28] Mbunkah HA, Marzel A, Schmutz S, Kok YL, Zagordi O, Shilaih M, et al. Low prevalence of transmitted HIV-1 drug resistance detected by a dried blood spot (DBS)-based next-generation sequencing (NGS) method in newly diagnosed individuals in Cameroon in the years 2015-16. J Antimicrob Chemother. 2018. 10.1093/jac/dky103 29635462

[29] Minister of Public Health (2015). National Guidelines for the Management of HIV/AIDS in Cameroon. https://aidsfree.usaid.gov/sites/default/files/cameroon_art_2015.pdf. Accessed on 23^rd^ January 2016.

[30] Machnowska P, Hauser A, Meixenberger K, Altmann B, Bannert N, Rempis E (2017). Decreased emergence of HIV-1 drug resistance mutations in a cohort of Ugandan women initiating option B+ for PMTCT. PLoS One.

[31] Wensing AM, Calvez V, Günthard HF, Johnson VA, Paredes R, Pillay D (2017). 2017 update of the drug resistance mutations in HIV-1. Topics in Antiviral Medicine.

[32] Teto G, Tagny CT, Mbanya D, Fonsah JY, Fokam J, Nchindap E (2017). Gag P2/NC and pol genetic diversity, polymorphism, and drug resistance mutations in HIV-1 CRF02_AG- and non-CRF02_AG-infected patients in Yaounde, Cameroon. Sci Rep.

[33] De Luca A, Sidumo ZJ, Zanelli G, Magid NA, Luhanga R, Brambilla D (2017). Accumulation of HIV-1 drug resistance in patients on a standard thymidine analogue-based first line antiretroviral therapy after virological failure: implications for the activity of next-line regimens from a longitudinal study in Mozambique. BMC Infect Dis.

